# Practical Strategies for Management of Lenalidomide-Associated Cytopenias in Myelodysplastic Syndromes With del(5q)

**Published:** 2017-11-01

**Authors:** Sandra E. Kurtin, Jean A. Ridgeway, Sara Tinsley

**Affiliations:** 1 University of Arizona and Arizona Cancer Center, Tucson, Arizona;; 2 University of Chicago Medical Center, Chicago, Illinois;; 3 H. Lee Moffitt Cancer Center and Research Institute, Tampa, Florida

## Abstract

**CASE STUDY**

A male patient aged 67 years with a 2-year history of refractory anemia and myelodysplastic syndromes (MDS) with del(5q) started lenalidomide (Revlimid) treatment as a participant in the MDS-001 trial (List et al., 2005). At the time of the study, this patient had been transfusion-dependent since 2001, and at study entry he had received a total of 12 units of red blood cells (RBCs). The patient started lenalidomide at 25 mg daily for 21 days of each 28-day cycle on April 2, 2002. (Please note that as a result of subsequent trials, the approved starting dose for lenalidomide in patients with del[5q] MDS is 10 mg.) The patient developed treatment-related pancytopenia in the first 3 weeks of treatment (see Figure on next page).

On day 24, lenalidomide was withheld. Platelet and white blood cell (WBC) counts were recovered during a 21-day period, and treatment was resumed with lenalidomide at 10 mg daily. During this initial dose interruption, the patient’s hemoglobin level improved. He achieved RBC-transfusion independence (TI) during week 5 of treatment—his last RBC transfusion was on April 22, 2002.

Bone marrow (BM) analysis, including fluorescence in situ hybridization, after 3 months of therapy, did not show the del(5q) abnormality. A repeat BM analysis after 57 months of treatment revealed minimal residual dyspoiesis, normal metaphase cytogenetics, and normal cell morphology. Subsequent BM biopsies showed transient trisomy 8 abnormalities but no del(5q) abnormality. The patient required one additional dose reduction during the first year of treatment. The patient did not require hospitalization during lenalidomide treatment but did have a history of seasonal allergies and a propensity for acute sinusitis. He received a single dose of pegfilgrastim (Neulasta) and a short course of antibiotics for active infections, generally one course of antibiotics per year.

This patient continued on lenalidomide at 5 mg daily for 21 days of every 28-day cycle without further dose reduction. The patient achieved durable RBC-TI and continued to receive treatment for more than 11 years. The levels of hemoglobin, platelets, and WBCs for an 11-year period in this patient are shown in the Figure. This patient had one normal platelet count in 11 years of lenalidomide treatment but no bleeding episodes. The average platelet count in the 11-year period was 67 × 1,000/∝L (normal range, 150–425 × 1,000/∝L). Similarly, the WBC count remained below normal, with an average of 3.1 × 1,000/∝L (normal range, 3.4–10.4 × 1,000/∝L). He remained active and continued working as an aerospace engineer until age 75.

This case demonstrates how effective management of cytopenias, through dose interruptions and modifications in the early weeks of treatment, helps to enable long-term lenalidomide treatment and a high quality of life. Despite the persistence of moderate, asymptomatic cytopenias, the patient was able to remain on lenalidomide therapy and maintained RBC-TI for more than 11 years. The patient died on October 4, 2014, at age 79.5, due to coronary artery disease and heart failure.

A heterogeneous group of bone marrow (BM) failure disorders, myelodysplastic syndromes (MDS) is common in older adults. Sustained unilineage or multilineage cytopenia is included in the mandatory diagnostic criteria of MDS (Valent et al., 2007). Anemia and transfusion dependence are inevitable in the majority of patients, and hematologic improvement (HI) and transfusion independence (TI) remain primary endpoints of clinical trials incorporating disease-modifying therapies (DMTs). It is important to note that achieving HI and TI may require up to 4 months of consistent DMT. Thrombocytopenia and neutropenia, the most common adverse events (AEs) associated with DMT, are prevalent in the first 8 to 12 weeks of treatment. Thus, strategies to monitor and manage cytopenias in patients with MDS are critical to patient safety and to enable adequate duration of therapy to evaluate efficacy.

**Figure 1:**
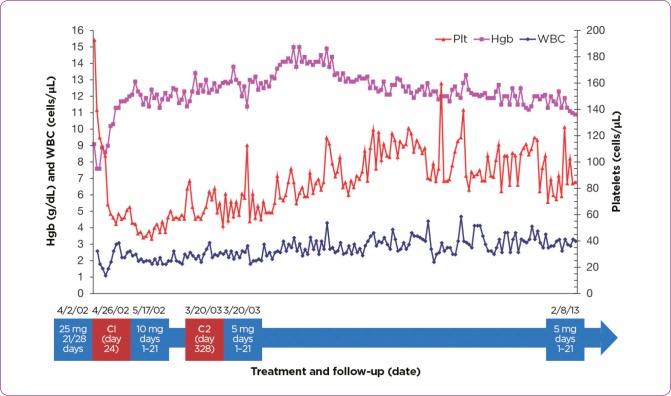
Levels of hemoglobin, platelets, and white blood cell counts for an 11-year period in the case study patient. C1 = cycle 1; C2 = cycle 2; Hgb = hemoglobin; Plt = platelets; WBC = white blood cell.

Currently three DMTs are approved by the US Food and Drug Administration (FDA) for treatment of MDS, including two hypomethylating agents, azacitidine and decitabine, and the immunomodulatory agent, lenalidomide (Revlimid). The only oral DMT available for the treatment of MDS is lenalidomide. The currently FDA-approved starting dose for lenalidomide in patients with del(5q) MDS is 10 mg. This article focuses on the monitoring and management of lenalidomide-associated cytopenias expected in patients with MDS with del(5q).

## LENALIDOMIDE IN THE TREATMENT OF MDS

Lenalidomide is an oral immunomodulatory drug that is licensed in the United States for patients with transfusion-dependent anemia due to International Prognostic Scoring System (IPSS) Low- or Intermediate-1 (Int-1)–risk MDS with del(5q) with or without additional cytogenetic abnormalities. In Europe, lenalidomide is licensed by the European Medicines Agency (n.d.) for the treatment of patients with transfusion-dependent anemia due to Low- or Int-1–risk MDS associated with del(5q) when other therapeutic options are insufficient or inadequate.

Although lenalidomide is associated with achievement of durable red blood cell (RBC)-TI in patients in the MDS-003 and MDS-004 trials (Fenaux et al., 2011; List et al., 2006), the most common AEs associated with lenalidomide treatment are neutropenia and thrombocytopenia. Familiarity with the incidence and time to onset of lenalidomide-related cytopenias, together with strategies for management, may help to realize the maximum potential benefit of therapy.

## STUDY DETAILS FROM MDS-003 AND MDS-004

Data from MDS-003 and MDS-004, and real-world patient experience, were used in summarizing the incidence of expected lenalidomide-associated cytopenias and in outlining strategies for practical management of cytopenias in patients with MDS. MDS-003 was a phase II, multicenter, single-arm study of 148 patients with lower-risk MDS (LR-MDS) and del(5q; List et al., 2006). Patients received 10 mg of lenalidomide daily for 21 days in 28-day cycles (n = 46) or as a 10-mg continuous daily dose (n = 102). MDS-004 was a phase III, multicenter, randomized, placebo-controlled, double-blind study of 138 patients with LR-MDS and del(5q; Fenaux et al., 2011). Patients were randomized to receive 10 mg of lenalidomide daily for 21 days in 28-day cycles (n = 69), a 5-mg continuous daily dose (n = 69), or a continuous daily dose of placebo (n = 67).

The incidence, time to onset, and recovery from cytopenias, as well as the incidence of lenalidomide dose modifications and discontinuations due to neutropenia or thrombocytopenia, were examined in patients from MDS-003 and MDS-004. Data were compared with long-term outcomes in the Case Study patient with LR-MDS and del(5q) who participated in the dose-finding MDS-001 trial (List et al., 2005). These data and expert experience were used to develop a practical approach to the management of cytopenias in lenalidomide-treated patients with MDS with del(5q).

## EXPECTED CYTOPENIAS: DATA FROM MDS-003 AND MDS-004

The most common grade ≥ 3 AEs in 286 patients treated with lenalidomide in the MDS-003 and MDS-004 trials were neutropenia (64%) and thrombocytopenia (41%; Fenaux et al., 2011; List et al., 2006). Grade ≥ 3 neutropenia and thrombocytopenia occurred most frequently during the first two treatment cycles (Fenaux et al., 2011; Giagounidis et al., 2008; List et al., 2006). In MDS-003, 62% of grade ≥ 3 hematologic AEs occurred in the first two treatment cycles (List et al., 2006). The median time to onset of grade ≥ 3 neutropenia in the study was 42 days (range, 14–411 days), and the documented median time to recovery was 17 days (range, 2–170 days; Celgene Corporation, 2017). The median time to onset of thrombocytopenia was 28 days (range, 9–290 days), and the documented median time to recovery was 22 days (range, 5–224 days; Celgene Corporation, 2017). It is important to note that patients with severe neutropenia (< 500 polymorphonuclear neutrophils/mm³) or severe thrombocytopenia (platelet count < 50,000/mm³) were excluded from MDS-003 (List et al., 2006), and patients with abnormal blood cell counts, including those with an absolute neutrophil count < 500/∝L or a platelet count < 25,000/∝L, were excluded from MDS-004 (Fenaux et al., 2011). 

In both trials, cytopenias were managed according to trial protocols, with dose interruptions of up to 21 days until cytopenias resolved to grade ≤ 2 and subsequent dose reductions when treatment was reinitiated. Thrombocytopenia and neutropenia were the most common reasons for lenalidomide dose modifications (Table 1; Fenaux et al., 2011; List et al., 2006). Treatment discontinuations due to neutropenia or thrombocytopenia in MDS-004 were infrequent relative to the incidence of these AEs, which suggests management with dose interruption and subsequent dose reduction is effective (Fenaux et al., 2011). 

**Table 1:**
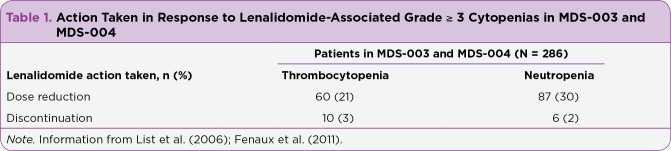
Action Taken in Response to Lenalidomide-Associated Grade ≥ 3 Cytopenias in MDS-003 and MDS-004

In MDS-003 and MDS-004, use of myeloid growth factors (MGF) was permitted for the management of neutropenia (Fenaux et al., 2011; List et al., 2005, 2006). Routine use of MGF to prevent infection is not recommended for patients with MDS (National Comprehensive Cancer Network [NCCN] Clinical Practice Guidelines, 2016), however, for patients with active infections or neutropenic fever, MGF, granulocyte colony-stimulating factors filgrastim (Neupogen) and pegfilgrastim (Neulasta), can be safely administered to patients concurrently with lenalidomide and might limit the severity of infections and the need for repeated dose reductions of lenalidomide (Kurtin & Sokol, 2006). 

In MDS-003, three deaths were attributed to neutropenic infection, which demonstrates the importance of closely monitoring patients for AEs and the potential role of MGF and other supportive care measures to alleviate neutropenia and infections (List et al., 2006). In MDS-004, 39 patients (57%) who received 10 mg of lenalidomide and 38 patients (55%) who received 5 mg of lenalidomide were given MGF (Fenaux et al., 2011). There were no deaths due to neutropenic infection, which may be the result of better monitoring and management of neutropenia. Concurrent use of MGF to treat neutropenia had no significant effect on the response to lenalidomide (Fenaux et al., 2011).

In MDS-003, treatment-emergent thrombocytopenia in patients with del(5q) MDS on lenalidomide was associated with an increased probability of achieving RBC-TI (Sekeres et al., 2008). By contrast, thrombocytopenia at baseline was associated with a low probability of patients achieving RBC-TI. Patients with baseline thrombocytopenia had significantly fewer consecutive days of lenalidomide treatment than those with treatment-emergent thrombocytopenia, as dose interruptions were required to manage thrombocytopenia (List et al., 2006). In patients with baseline thrombocytopenia, low exposure to lenalidomide may reduce the probability of an erythroid response. In addition, patients with a normal platelet count at the start of treatment who develop thrombocytopenia may go on to have a favorable erythroid response, emphasizing the importance of effective management strategies and continued treatment.

The mean time to response in patients with MDS and del(5q) treated with lenalidomide was variable. In MDS-003, the median time to RBC-TI was 4.6 weeks (range, 1–49 weeks; List et al., 2006). In MDS-004, the onset of an erythroid response occurred in 48.8% of patients during cycle 1, 37.2% of patients during cycle 2, 9.3% of patients during cycle 3, and 4.7% of patients during cycle 4 (Fenaux et al., 2011). This variation in the time to response emphasizes the need for an adequate trial length (minimum of 4 months) to achieve maximal benefit in the absence of intolerance or unacceptable drug toxicity. Early discontinuation of treatment, unfortunately, remains common, essentially exposing patients to drug toxicity without the full potential for benefit (Kurtin, 2011). Continuation of therapy until unacceptable toxicity or disease progression is recommended in patients who respond to lenalidomide (Celgene Corporation, 2017; Fenaux et al., 2011; List et al., 2006).

## PRACTICAL GUIDANCE FOR MANAGEMENT OF CYTOPENIAS

Perhaps the most important factor to ensure an adequate clinical trial of lenalidomide is to set expectations that 4 months of treatment are needed to adequately evaluate response. Establishment of expectations at the outset of treatment, together with a formal plan to monitor blood cell counts and tolerance, is essential to maintain treatment in the first 12 weeks. This level of care requires a partnership between the health-care provider (HCP), the patient, and the patient’s caregivers.

Weekly blood cell counts are necessary in the first 8 weeks. The results must be reviewed and acted upon to improve early recognition of cytopenias, enable prompt management, and minimize the severity of complications. Patients treated with lenalidomide should be educated about the signs and symptoms that warrant making contact with an HCP, including who to call and when. The recommended management of lenalidomide-related neutropenia and thrombocytopenia is available in the lenalidomide package insert (Celgene Corporation, 2017). Advice is given on dose interruptions and dose reductions dependent upon criteria such as the initial lenalidomide dose, severity, and time of onset of cytopenias.

Summaries of the guidelines for the management of cytopenias within the first 4 weeks and after 4 weeks of lenalidomide treatment are shown in Tables 2 and 3, respectively. Guidelines may need to be tailored to individual patients based on their BM reserve, proximity to the treatment center, and availability of transfusion services and emergency care services (Kurtin, Demakos, Hayden, & Boglione, 2012). Additional tips for management of cytopenias in this patient population, gleaned from trial reports and clinical experience, are summarized in Table 4.

**Table 2:**
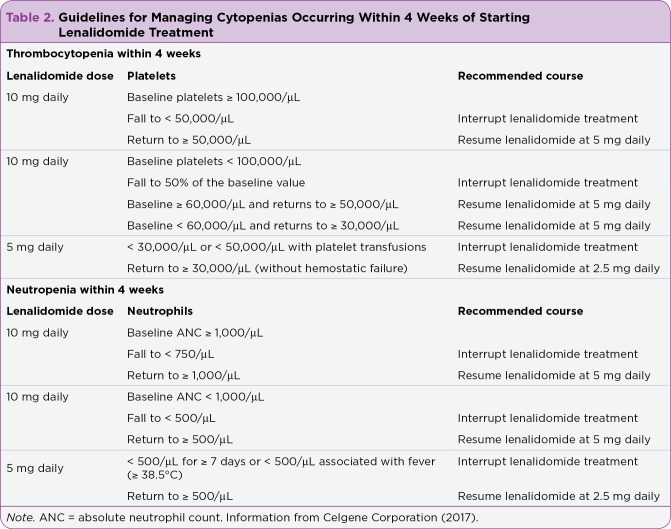
Guidelines for Managing Cytopenias Occurring Within 4 Weeks of Starting Lenalidomide Treatment

**Table 3:**
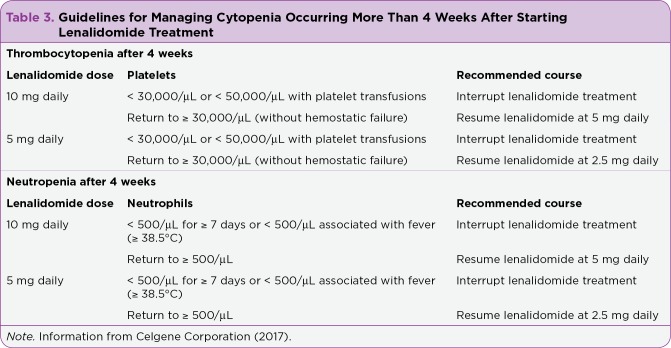
Guidelines for Managing Cytopenia Occurring More Than 4 Weeks After Starting Lenalidomide Treatment

**Table 4:**
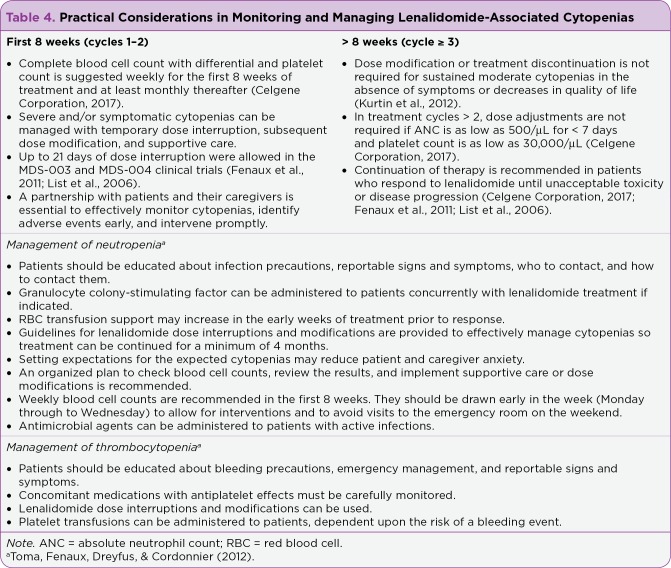
Practical Considerations in Monitoring and Managing Lenalidomide-Associated Cytopenias

Adverse events (bacteremia and mucositis) seen in patients with cytopenias and acute myeloid leukemia are much less common in patients with Low-/Int-1–risk MDS and in many patients with Int-2–risk MDS receiving DMTs (Kurtin, 2011). It is also important to remember that several commonly used medications, including some over-the-counter medications, can cause cytopenias. Caution is therefore warranted when patients use these agents concomitantly with lenalidomide. Examples of common medications that can exacerbate thrombocytopenia include anti-inflammatory drugs (e.g., aspirin, ibuprofen, naproxen, acetaminophen), antimicrobial agents (cephalosporins, penicillins), and anticoagulants (warfarin, dalteparin [Fragmin], rivaroxaban [Xarelto]; Warkentin, 2013).

## DISCUSSION

Most responses to lenalidomide occur rapidly after treatment initiation; however, it may take up to four treatment cycles (16 weeks) to achieve HI and RBC-TI (Fenaux et al., 2011). At least four cycles of treatment with lenalidomide are recommended in patients with del(5q) MDS to adequately evaluate response (Kurtin & Sokol, 2006; List et al., 2005). Cytopenias, specifically neutropenia and thrombocytopenia, are the most common AEs reported in patients with LR-MDS and del(5q) receiving lenalidomide (Celgene Corporation, 2017; Fenaux et al., 2010, 2011; List et al., 2006) and are the most common in the first 8 weeks of treatment. Blood counts, therefore, should be monitored weekly during this period to mitigate risks and institute the recommended dose modifications and supportive care strategies. Continuation of therapy until unacceptable toxicity or disease progression based on the International Working Group criteria (Cheson et al., 2006) is recommended for patients who achieve HI or TI.

Sustained, moderate, but asymptomatic cytopenias are common in patients being treated with lenalidomide and other DMTs for MDS (Kurtin & Sokol, 2006; Kurtin & List, 2009). In the absence of acute symptoms (e.g., fever, chills, active infection, bleeding), moderate cytopenias do not require dose reduction or discontinuation of therapy. Most patients who develop symptomatic or more severe cytopenias can be effectively managed with dose modifications and supportive care, which avoids premature discontinuation of therapy and improves the potential to achieve maximum benefit (Celgene Corporation, 2017; Fenaux et al., 2010, 2011; Kurtin et al., 2012; List et al., 2006). Our case study of a patient who received lenalidomide for 11 years with sustained RBC-TI illustrates how moderate but asymptomatic neutropenia and thrombocytopenia do not require dose interruptions and may be managed without the need for continued lenalidomide dose modifications or discontinuation.

Currently only three FDA-approved agents are available for the treatment of MDS; as two of these agents are similar drugs, it is imperative to maximize the use of each one. Establishment of expectations, organization of a plan for weekly laboratory tests with a review of results and follow-up visits, and utilization of dose interruptions and reductions in the early weeks of treatment provide practical strategies to manage cytopenias in patients receiving lenalidomide for del(5q) MDS that will enable them to continue therapy and achieve optimal clinical benefit.
